# Multiple Automated Health Literacy Assessments of Written Health Information: Development of the SHeLL (Sydney Health Literacy Lab) Health Literacy Editor v1

**DOI:** 10.2196/40645

**Published:** 2023-02-14

**Authors:** Julie Ayre, Carissa Bonner, Danielle M Muscat, Adam G Dunn, Eliza Harrison, Jason Dalmazzo, Dana Mouwad, Parisa Aslani, Heather L Shepherd, Kirsten J McCaffery

**Affiliations:** 1 Sydney Health Literacy Lab, Sydney School of Public Health Faculty of Medicine and Health The University of Sydney Sydney Australia; 2 Biomedical Informatics and Digital Health Faculty of Medicine and Health The University of Sydney Sydney Australia; 3 Western Sydney Local Health District Health Literacy Hub Sydney Australia; 4 School of Pharmacy Faculty of Medicine and Health The University of Sydney Sydney Australia; 5 Susan Wakil School of Nursing and Midwifery Faculty of Medicine and Health The University of Sydney Sydney Australia

**Keywords:** health literacy, comprehension, health education, health communication, medicine information, readability

## Abstract

Producing health information that people can easily understand is challenging and time-consuming. Existing guidance is often subjective and lacks specificity. With advances in software that reads and analyzes text, there is an opportunity to develop tools that provide objective, specific, and automated guidance on the complexity of health information. This paper outlines the development of the SHeLL (Sydney Health Literacy Lab) Health Literacy Editor, an automated tool to facilitate the implementation of health literacy guidelines for the production of easy-to-read written health information. Target users were any person or organization that develops consumer-facing education materials, with or without prior experience with health literacy concepts. Anticipated users included health professionals, staff, and government and nongovernment agencies. To develop this tool, existing health literacy and relevant writing guidelines were collated. Items amenable to programmable automated assessment were incorporated into the Editor. A set of natural language processing methods were also adapted for use in the SHeLL Editor, though the approach was primarily procedural (rule-based). As a result of this process, the Editor comprises 6 assessments: readability (school grade reading score calculated using the Simple Measure of Gobbledygook (SMOG)), complex language (percentage of the text that contains public health thesaurus entries, words that are uncommon in English, or acronyms), passive voice, text structure (eg, use of long paragraphs), lexical density and diversity, and person-centered language. These are presented as global scores, with additional, more specific feedback flagged in the text itself. Feedback is provided in real-time so that users can iteratively revise and improve the text. The design also includes a “text preparation” mode, which allows users to quickly make adjustments to ensure accurate calculation of readability. A hierarchy of assessments also helps users prioritize the most important feedback. Lastly, the Editor has a function that exports the analysis and revised text. The SHeLL Health Literacy Editor is a new tool that can help improve the quality and safety of written health information. It provides objective, immediate feedback on a range of factors, complementing readability with other less widely used but important objective assessments such as complex and person-centered language. It can be used as a scalable intervention to support the uptake of health literacy guidelines by health services and providers of health information. This early prototype can be further refined by expanding the thesaurus and leveraging new machine learning methods for assessing the complexity of the written text. User-testing with health professionals is needed before evaluating the Editor’s ability to improve the health literacy of written health information and evaluating its implementation into existing Australian health services.

## Introduction

Health literacy describes a person’s capacity to access, understand, appraise, and use information and services to promote and maintain good health [1]. National and international policies increasingly recognize disparities in health literacy as a critical source of health inequality. This was demonstrated most recently by the World Health Organization, which positioned health literacy as one of the 3 key pillars needed to achieve the Sustainable Development Goals [2]. A recent review demonstrated that internationally, policies concerning health literacy consistently argue that providing health information that all people can easily access and understand is fundamental to addressing health literacy [3].

However, integration of easy-to-understand health information into routine practice rarely happens, despite being a relatively simple and low-cost strategy. For example, less than 1% of web-based Australian health information is estimated to meet recommended grade reading levels [4]. This issue persists even in the face of a global pandemic, where timely dissemination of understandable information is extremely important. Analysis of international COVID-19 materials from governments and official sources indicates that, on average, these are written above the recommended Grade 8 level for the general population, making them unsuitable for people with low health literacy [5].

Several well-established health literacy guidelines provide advice about how to structure, write, and visually present health information, for example, the Universal Precautions Toolkit [6] and the Patient Education Materials Assessment Tool (PEMAT) [7]. One of the most widely used health literacy guidelines recommends writing health information at or below a Grade 8 reading level in countries such as Australia [4,8] or Grades 5 to 6 in the United States [9]. This is a useful standard because it is specific, objective, replicable, and readily available from web-based readability calculators. However, the concept of a grade reading score is narrow in scope, and many of the underlying formulas assess language complexity primarily in terms of syllable counts, the lengths of words, and the lengths of sentences [10]. Additional guidelines advise on other aspects of the text, including how common the words are, sentence structure, grammar, overall structure, and the flow of ideas in the text. The importance of these other criteria is supported by recent machine learning algorithms that predict the accessibility of health information. For example, these studies have shown that text features such as familiarity with the text’s vocabulary and cohesion across sentences may also play an important role [11-16].

However, to date, many of the health literacy guidelines do not afford the same level of specificity and objectivity as those for grade reading scores. This is illustrated through the example of a PEMAT item that advises the use of common, everyday language. Though this guideline is valuable, it can be challenging to implement as there are no detailed instructions on how to assess or act on this criterion, notably which words are considered “everyday” and what an acceptable number of uncommon words for a given text length might be [7].

With recent advances in computer science, web-based software may be able to address this issue. For example, Ondov and colleagues [17] recently identified 45 papers investigating simplification for biomedical texts, including 32 tools or methods. Of these, 22 tools took a procedural (rules-based) approach; 10 primarily used a machine learning approach, that is, through natural language processing. This is a rapidly developing area of research. The authors noted that, though machine learning approaches provide more sophisticated output than traditional grade reading scores, the quality of these models is currently constrained by the training data sets that are available. In contrast, the authors argue that procedural approaches, though likely to provide less tailored feedback, have the benefit of being more predictable.

Regardless, few of these projects have resulted in tools that can be easily accessed and used by health services staff. There are some existing web-based platforms that provide detailed feedback on general writing style. For example, the Hemingway App [18], Grammarly [19], StyleWriter [20], and VisibleThread [21] are web-based tools that variously provide feedback on aspects of the text such as readability (including long words and long sentences), unnecessary adverbs, passive voice, formality, tone, and engagement. However, only some of these provide specific suggested alternative phrasing to reduce the complexity of health information, with none specifically addressing health and medical jargon, such as terms identified by the Centers for Disease Control and Prevention’s Everyday Words for Public Health Communication [22]. Further, none have been specifically designed for health contexts or with health literacy guidelines in mind. Leroy and colleagues [23] have developed a promising tool for the simplification of medical information; however, the tool will require further testing to ensure it aligns with health literacy principles and establish whether it can be used effectively by health information developers.

In 2020, our team developed a web-based platform to broaden the range of automated assessments available to people developing patient-facing health information in a manner that is easy for health staff to understand and use. This paper outlines the development of the “SHeLL (Sydney Health Literacy Lab) Health Literacy Editor,” including the rationale and operationalization for each of the included objective assessments.

## Development of the SHeLL Health Literacy Editor

### Objectives of the SHeLL Health Literacy Editor

The SHeLL Health Literacy Editor aimed to assist Australian health information providers to develop health education materials for patients or community members (herein referred to as “consumers”) that adhere to health literacy guidelines to improve the quality, safety, and ease of reading of written health information. This would be achieved by developing objective and programmable health literacy assessments informed by existing health literacy guidelines (“inputs”) and established objective assessments from other fields, for example, linguistics. Where possible, other strategies to promote good health literacy practice were also incorporated, for instance, by providing immediate feedback on specific words, phrases, or sentences in addition to whole-text assessments such as readability.

### Target Users

We identified our target users as any person or organization that develops consumer-facing health information materials. We do not anticipate that users necessarily have prior experience with health literacy concepts, but would expect users to speak and understand English and to have sufficient skills to write health information in English. Users may include health professionals, staff, and government and nongovernment agencies.

### Inputs

Existing guidelines related to health literacy informed the selection of assessments in the SHeLL Health Literacy Editor (Table 1). Items from these guidelines were incorporated into the Editor if they were amenable to automated assessment, for instance, through calculations involving counts of the numbers of words and sentences, string (character) searches, or identification of grammar.

**Table 1:** Guidelines and resources informing SHeLL (Sydney Health Literacy Lab) Health Literacy Editor assessments.

Guideline or resource^a^	Description and scope	Items amenable to incorporation into the SHeLL Health Literacy Editor
Universal Precautions Toolkit [9]	A suite of 21 tools to promote health literacy in 4 domains: spoken communication, written communication, self-management and empowerment, and supportive systems	Tool #11 (Assess, select, and create easy-to-understand materials): specific relevant recommendation is to write at the 5th or 6th grade reading level
Patient Education Materials Assessment Tool [7]	Subjectively rated tool to assess the understandability and actionability of health information. For printed materials, this includes 17 items that assess understandability and 7 items that assess actionability.	Item 3: The material uses common, everyday language; Item 4: Medical terms are used only to familiarize the audience with the terms; Item 5: The material uses the active voice; Item 8: The material breaks or “chunks” information into short sections
Centers for Disease Control and Prevention Clear Communication Index [24]	20-item tool to improve public communication adherence to plain language guidelines and support the implementation of US health literacy policies	Item 6: Use active voice Item 7: Use words the primary audience uses Item 8: Chunk information
Evaluative Linguistic Framework [25]	Framework for assessing patient information leaflets, based on linguistic theory. Items include consideration of organization and structure, metadiscourse, headings, technicality of vocabulary, lexical density, the relationship between reader and writer, and format	Technicality of vocabulary; Lexical density
Plain Language [26]	Guidelines for preparing texts to meet US plain language standards, including text grade reading level, organization, and word choice.	Use simple words and phrases (for words that can be identified using a string search); avoid noun strings; avoid jargon; minimize abbreviations; use active voice; write short paragraphs; write short sentences
Health Literacy Online [27]	Guidelines for web-based health information, including writing actionable content, displaying content clearly, organizing content and simplifying navigation, engaging users, and user testing	2.6 (Write in plain language)
Everyday words for public health communication [22]	A thesaurus containing simpler alternatives to public health jargon	All entries
Simply Put: Writing and Design Tips [28]	Guidelines for preparing easy-to-understand information, including the written text, visual aspects, and testing with consumers	“Use everyday words,” “Keep sentences short,” “spell out acronyms,” “Use active verbs”
Suitability Assessment of Materials [29]	Subjectively rated tool to assess the suitability of health-related information for adults, including content, literacy demand, graphics, layout, learning stimulation and motivation, and cultural appropriateness	Literacy demand (Score of 5th Grade reading level or lower=superior; 6th-8th Grade=adequate; 9th Grade or above=not suitable).
Person-centered language [30-35]	Various language position statements from Australian peak bodies outlining preferred language for a given health condition	Words or phrases that could be identified using a string search
Question Understanding Aid [36]	Web-based tool to assess the comprehensibility of survey questions and response options.	Unfamiliar technical term, complex syntax, working memory overload

^a^Note: DISCERN [37] is a subjectively rated tool to assess the quality of consumer health information and treatment choices (eg, clearly stated aims, information sources, and descriptions of treatments). Though potentially relevant, no items were identified that could be incorporated into the SHeLL Health Literacy Editor.

### Functionality Considerations

As far as possible, the SHeLL Health Literacy Editor was designed to provide automated, immediate, and objective feedback on written health text. This was facilitated by incorporating software that can process and analyze English-language text called spaCy [38]. SpaCy breaks down text into sentences and words. It then uses rule-based methods and trained models to identify grammatical information about each word. This information includes the word’s part of speech (eg, whether it is a noun, preposition, or verb), lemma (base word form, eg, “write” is the lemma for the word “written”), and whether the word is a named entity (eg, John, Canada, Monday).

### Rationale for Including Assessments

#### Overview

Based on the above inputs (Table 1), we identified 6 assessments that could be implemented for real-time use while editing a document on a web-based interface: readability, complex language, passive voice, text structure, lexical density or diversity, and person-centered language. For each of these assessments, we describe the rationale for its inclusion below.

#### Readability

Readability estimates how difficult a text is to read, often presented in the form of a “Grade Reading Score” [9]. Grade reading scores are identified as a useful tool in many health literacy guidelines [8,9,39] and are widely used in health literacy research (see, eg, [5]). A variety of readability formulas are used to assess health information [40,41]. We identified the Simple Measure of Gobbledygook (SMOG) [42] as the most appropriate readability formula for the SHeLL Health Literacy Editor. It is the only readability formula for which the grade reading score assumes the reader has a complete comprehension of the text [40]. For example, the SMOG assumes that a Grade 8 reader would score 100% on a multiple-choice comprehension test for a text written at a Grade 8 reading level. By comparison, the Flesch Reading Ease assumes that Grade 8 readers would correctly answer 75% on a multiple-choice comprehension test for the same text [43]. The Flesch Kincaid, another widely used readability formula, assumes 35% comprehension based on a cloze test rather than multiple choice questions [44]. As such, the SMOG provides a more conservative estimate of the grade reading score than other common readability formulas [40,44,45]. Other studies have demonstrated that SMOG assessments are also more consistent across random sampling within a text and are less sensitive to differences in formatting [40]. A target of a Grade 8 reading score or lower was selected to match Australian recommendations [8].

#### Complex Language

All health literacy guidelines emphasize the need to use simple, everyday language and minimize medical jargon (Table 1). In some instances, medical terminology may be required and should be defined and explained in simpler words. Similarly, acronyms are also often considered technical terms that should be defined in the first instance [28].

#### Passive Voice

Using active voice is a key recommendation to improve how easy health information is to understand and act upon [7]. The passive voice refers to a grammatical construction that emphasizes the recipient of an action (eg, “the blood test was ordered by the doctor”), whereas the active voice places an emphasis on the entity carrying out the action (“the doctor ordered the blood test”).

#### Text Structure

**Figure 1:**
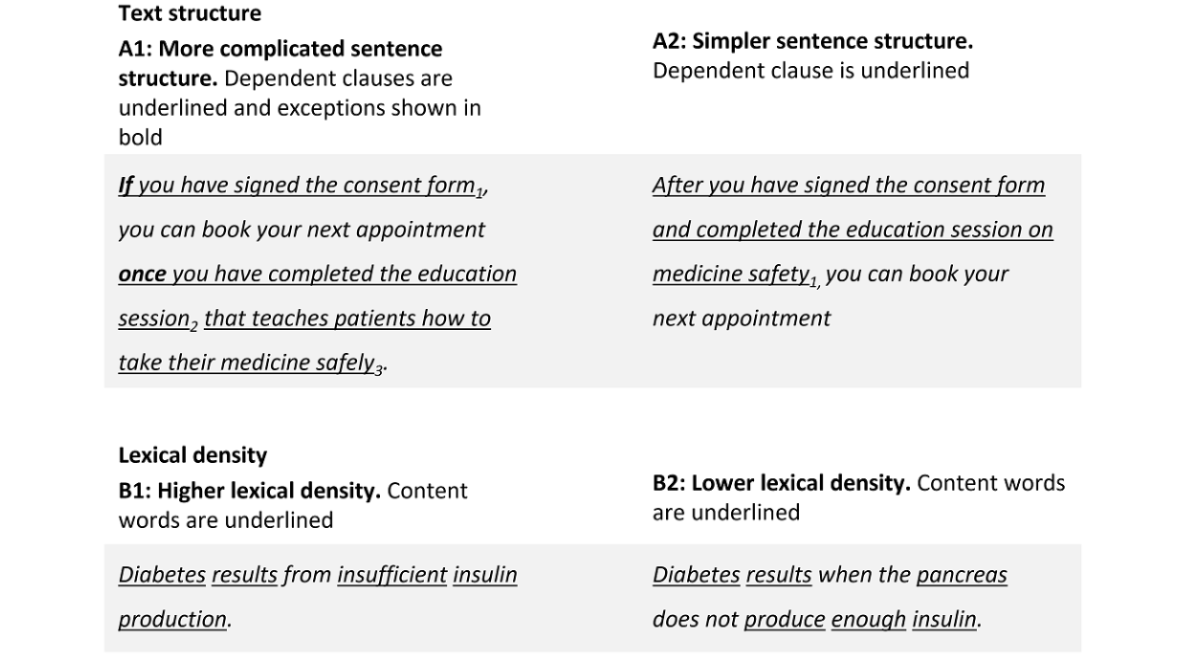
Illustrative examples of text structure (Panels A1 and A2) and lexical density (Panels B1 and B2). Simpler alternatives are shown in Panels A2 and B2. These examples are intended to illustrate differences in text structure and lexical density, respectively. Texts A2 and B2 may benefit from further simplification, for example, using dot points for each step in A2 and using simpler words in B2.

The structure of paragraphs and sentences was identified as a factor relevant to text complexity by several guidelines (Table 1). For example, Health Literacy Online recommends keeping paragraphs to 3 lines or less [27]. Similarly, the US Plain Language guidelines recommend that paragraphs be between 3 and 8 sentences long and no more than 150 words [26]. The Plain Language guidelines also advise against “sentences loaded with dependent clauses and exceptions” [26]. An example is depicted in Panel A1 of Figure 1, in which 3 dependent clauses are underlined and numbered. The text can be restructured to improve clarity by reducing the number of dependent clauses and replacing words that indicate exceptions (Figure 1, Panel A2).

Lastly, the Question Understanding Aid’s “Working Memory Overload” assessment (Table 1; [36]) advises against double-barreled phrasing and convoluted questions. For example, “Do you think that diet and exercise are effective for managing diabetes and cardiovascular disease?” is a double-barreled question. Responses could variously refer to diet, exercise, or both types of interventions and may relate to diabetes, cardiovascular disease, or both conditions.

#### Lexical Density and Diversity

Lexical density is a component of the Evaluative Linguistic Framework (Table 1; [25]). However, lexical density and diversity have not been extensively studied in health contexts despite being common computational linguistic assessments [14,46]. Lexical density measures the ratio of words in a text that are “content words” versus “function words.” Content words tell us what a text is about (nouns, adjectives, most verbs, and most adverbs). Function words are those that carry grammatical meaning. A text with higher lexical density, therefore, conveys meaning more concisely. For example, compare the sentences in Panels B1 and B2 of Figure 1. The sentence in Panel B1 has a higher lexical density, with a ratio of 5 content words:1 function word, compared to the sentence in Panel B2 (6 content words:4 function words). Conceptually, it may be beneficial for health information materials to have a lower lexical density, as this style of writing is more indicative of spoken English (usually with a lexical density score between 1.5 and 2) than written English (usually between 3 and 6) [47]. This aligns with health literacy guidelines that recommend writing with a “conversational tone” [26,27].

Lexical diversity measures the proportion of words in a text that are unique. Higher lexical diversity indicates that a text has a larger vocabulary [48]. A text with higher lexical diversity may use more words for the same concept, for example, “cancer,” “carcinoma,” and “neoplasm.” A text with low lexical diversity may simply refer to “cancer.”

#### Person-Centered Language

It is widely recommended that health information adopt a person-centered approach to health services [49,50]. Language can have a lasting impact on how people understand their condition, their treatment, and their place in the community. Person-centered language seeks to reduce blame, stigma, and judgment and encourage accuracy, autonomy, respect, and inclusion [51].

### Operationalization of Assessments

#### Readability

**Figure 2:**
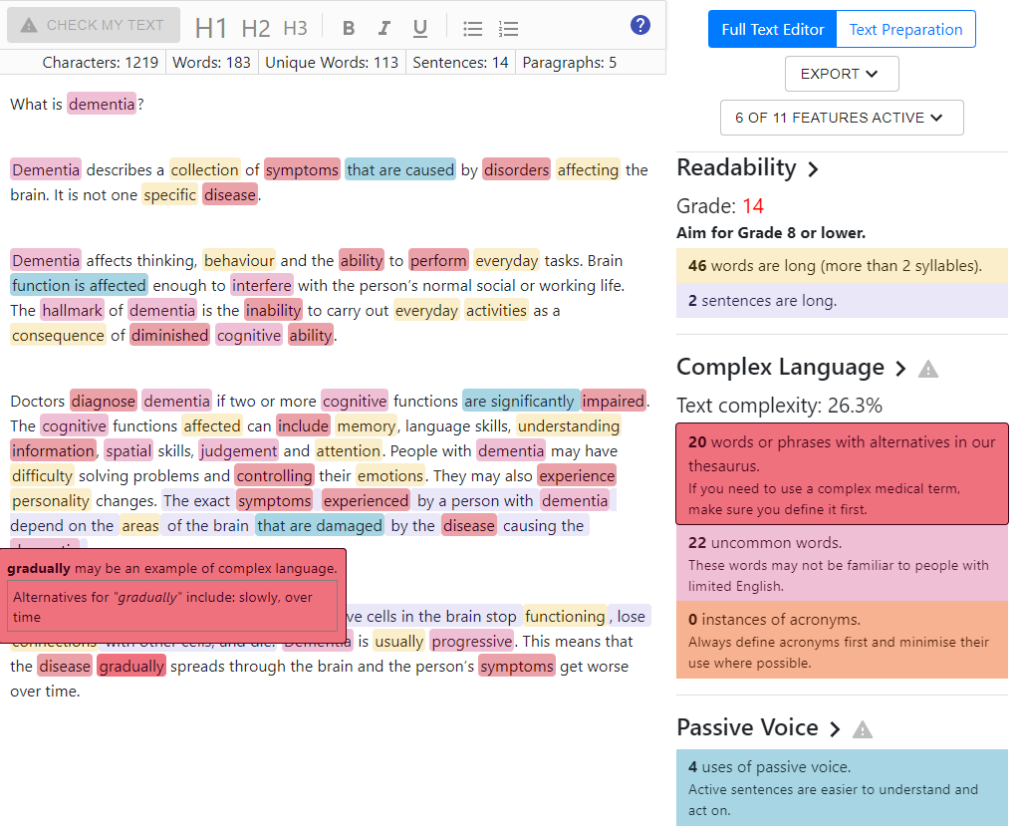
Screenshot of the SHeLL (Sydney Health Literacy Lab) Health Literacy Editor v1, full-text editor pane.

The SHeLL Health Literacy Editor provides an overall Grade Reading Score based on the SMOG formula, rounded to the nearest whole number (Figure 2). The SMOG formula estimates the Grade Reading Score based on the proportion of words in each sentence that are multisyllabic (>2 syllables). The Editor counts the number of syllables using an open-source English language dictionary that provides syllable counts for over 115,000 words [52]. If a given word is not listed in the dictionary, the syllable count is estimated from the patterns of vowels and consonants. To ensure the accuracy of the SMOG score presented to users, the automated calculation was compared to manually calculated scores using prose text that did not contain ambiguous syllable counts (eg, numbers and acronyms that can be pronounced as individual letters or as a single word, eg, “WHO” for World Health Organization).

To assist users, the SHeLL Health Literacy Editor flags words in the text that are contributing to a higher SMOG calculation (ie, words that are >2 syllables). The Editor also flags sentences longer than 20 words. This sentence length was selected on the basis of other health literacy recommendations [27].

#### Complex Language (Vocabulary)

We identified several resources that provide simpler alternatives to complex language, including the Centers for Disease Control and Prevention’s Everyday Words for Public Health Communication, which was developed specifically to address health literacy needs in health communication [22]. Thesaurus entries from these resources were collated into a database listing the word, relevant string searches, and an accompanying thesaurus entry containing possible alternatives. Users can access thesaurus entries by hovering over a word (Figure 2).

Users can enter up to 5 words that will be excluded from the complex language assessment if they believe readers will be familiar with the terms. This feature affords flexibility to the user while also seeking to discourage users from exempting *all* jargon from the complex language assessment. The maximum number of excluded words will be further refined as user feedback is gathered.

In addition, the Editor identifies words that are uncommon in the English language based on word frequencies in a database of more than 270 million words from diverse English-language sources (learner materials, fiction, journals and magazines, nonfiction, radio, spoken English, documents, and TV) [53]. The database was specifically designed to identify words that would be most useful to people learning English as a second language. For example, its authors claim that the most frequent 2800 words provide learners with 90% coverage for general English texts [53]. This assessment also uses spaCy’s trained named entity recognition model to prevent named entities such as companies, locations, organizations, languages, countries, and periods of time from being flagged as uncommon.

Acronyms were identified as a series of at least 2 capital letters, or capital letters with a period in between. Lowercase letters were allowable as this is common practice in health (eg, SHeLL for Sydney Health Literacy Lab).

An overall “text complexity” score is calculated from the proportion of words flagged with any of the 3 complex language assessments (“thesaurus,” “acronyms,” or “uncommon words”). No targets were available as this is a new objective assessment.

#### Passive Voice

The SHeLL Health Literacy Editor identifies patterns of the verb “to be” (eg, “is,” “were”) and a past participle (eg, “delivered,” “given”) that indicate passive voice. Users can read a brief description of the passive voice, including worked examples that change passive voice constructions into the active voice.

#### Text Structure

The SHeLL Health Literacy Editor provides guidance on paragraph length by flagging paragraphs that are longer than 8 sentences or more than 150 words. This criterion also aligns with recommendations from the US Plain Language guidelines [26].

The Editor identifies complex questions as those consisting of at least 12 words and more than 2 conjunctions (“for,” “and,” “nor,” “but,” “or,” “yet,” and “so”), based on the Question Understanding Aid’s “Working Memory Overload” assessment (Table 1; [36]). In doing so, this flag aims to identify potential instances of double-barreled or convoluted questions but should only be considered a proxy for complex questions.

#### Lexical Density and Diversity

The SHeLL Health Literacy Editor uses information about the part of speech to determine whether a word fulfills a function or content role. Prepositions (eg, in, on), pronouns (eg, she, them), determiners (eg, the, a), conjunctions (eg, and, that), and auxiliaries (eg, is, got, do) are categorized as function words; all other parts of speech are categorized as content words. The ratio of content words to function words per clause is then calculated [47].

The SHeLL Health Literacy Editor computes an unstandardized and standardized assessment of lexical diversity. The unstandardized assessment, or “type-token ratio,” is the ratio of unique words to total words. The type-token ratio is correlated with text length [14]. The Measure of Lexical Textual Diversity [54] is a standardized type-token ratio that adjusts for text length by averaging the type-token ratio across sequential strings of words within the text. Measure of Lexical Textual Diversity is more stable across texts of different lengths [54,55].

#### Person-Centered Language

The SHeLL Health Literacy Editor draws on peak-body guidance for person-centered language across several conditions: diabetes, dementia, chronic pain, cancer, and mental health (including language that aligns with trauma-informed care) [30-35]. As language guidelines become available for other health conditions, these can be incorporated into the Editor. This feature flags sections of text that contain easily identifiable examples of language that are not person-centered; for example, rather than “sufferer,” guidelines recommend referring to “a person living with X condition.” Of note, this feature is not comprehensive, as some aspects of person-centered guidelines require the writer to consider aspects that are broader than individual words or phrases that can be identified using a string search function.

### Usability

#### Overview

We implemented 4 features to assist with usability: a “text preparation” mode; ordering the assessments by importance (a hierarchy of assessments); functions to export the revised text to a Word document; and exporting a summary of assessments as a PDF. In addition, where possible, user instructions and feedback have been framed to set clear expectations about the intended use of the Editor and its assessments.

#### Text Preparation

Preparing a text for readability assessment is an important aspect of calculating a grade reading score. However, this preparation can be cumbersome when text (eg, headings) must be removed or altered for assessment purposes but is ultimately included in the document. To reduce this burden, the SHeLL Health Literacy Editor allows users to indicate which segments of text to exclude from the readability calculation without having to edit the text itself. Common text preparation decisions are set as a default setting [41]. For example, by default, the Editor does not count short bullet points (less than 4 words), headings that are less than 4 words, or URLs. Bullet points are considered a “sentence” even if there is no full stop at the end.

#### Hierarchy of Assessments

The SHeLL Health Literacy Editor flags sections of the text using opaque rectangular boxes (“highlights”) of different colors (Figure 1). Each color represents a different assessment. Assessments that are higher priorities overlay those that are lower priorities. This hierarchy prioritizes guidance for complex language, followed by passive voice, readability, and complex structure. Users can toggle assessments on or off to view overlapping highlights. To avoid overwhelming new users, only the 3 highest-ranked assessments are active by default: complex language, passive voice, and readability.

#### Export and Summary Features

Users can export a copy of the text as a Word document or as a “summary file” that provides all objective assessments and information about text preparation decisions, including the maximum of 5 words excluded from the complex language assessments.

#### Setting Expectations for Intended Use

We anticipate that users may need guidance to correctly interpret Editor feedback. For example, there is a risk that users may feel the need to remove all highlights from the text for the simplification task to be considered “complete.” To mitigate frustration and set realistic expectations, the Editor’s prompts and instructions emphasize that there are likely to be some highlighted words and, rather, to aim to make the text as simple as possible (eg, Aim for Grade 8 or lower).

## Discussion

### Summary

The SHeLL Health Literacy Editor is an urgently needed, innovative tool to support the timely development of health-literate written health information. It objectively assesses the readability, complex language, passive voice, text structure, lexical density and diversity, and person-centered language. By explicitly aligning features with existing health literacy guidelines, the tool provides health information developers with a unique and targeted tool to improve the quality and safety of health information. The fact that assessments are provided in real-time supports iterative revisions. to reduce text complexity.

A key strength of the SHeLL Health Literacy Editor is that it complements the widely and almost exclusively used readability score with other relevant assessments, including those specific to health. Other strengths include its capacity to improve the efficiency of preparing texts for readability analyses through the text preparation function; its capacity to build workforce skills in applying health literacy principles; and its feasibility for scaling up across an organization or jurisdiction given the minimal cost and resources involved. We have also completed extensive user testing of the SHeLL Health Literacy Editor with health staff, which is reported separately (Ayre et al, unpublished data). User-testing sought to evaluate and improve acceptability and usability, help prioritize additional features, and identify training needs.

It is important to emphasize that the SHeLL Health Literacy Editor does not replace more comprehensive health literacy guidelines. For example, the PEMAT also provides guidance on actionability and visual elements. We envisage its scope as assisting people to develop simpler *text* to convey health information. A few specific aspects of the written text are also outside its scope. For example, strategies for communicating risk accurately and without bias [56] and guidelines about written text that operate beyond the level of the sentence (eg, outlining the text’s purpose and logical sequence of information) are also largely outside the current scope of the Editor, though they could be considered in future iterations.

We envision that the SHeLL Health Literacy Editor would be used in the early stages of resource development. Involving consumers is critical to developing accessible and understandable health information resources [57]. However, obtaining consumer feedback is resource-intensive. The Editor will facilitate an efficient and scalable process in which health literacy principles are applied as much as possible to a text prior to consumer involvement. The Editor may also improve translation efforts by ensuring that the parent text is expressed simply prior to translation.

### Future Directions

There are many avenues for further research involving the Editor. We intend to evaluate the Editor’s ability to improve the health literacy of written health information and evaluate its implementation into existing Australian health services. This evaluation could also investigate the relative importance of each of the Editor’s assessments and establish appropriate objective health literacy benchmarks that would complement existing subjective health literacy guidelines.

Currently, the Editor’s features take a primarily procedural (rules-based) approach. In future iterations, increased use of machine learning approaches could enhance the Editor’s features. For example, the Editor could highlight sentences containing many dependent clauses and give specific advice about how to simplify these sentence structures. As another example, the value of the thesaurus function is largely driven by the number, quality, and relevance of the thesaurus entries. This could be further enhanced by leveraging large existing (manually developed) medical dictionaries and by incorporating machine learning methods that have “mined” pairs of jargon and lay terms using multiple corpora [17,58-60]. The uncommon language feature may be further improved by using the “SciSpaCy” variant that has been adapted to biomedical texts, as this may result in improved identification of medically named entities.

Beyond the structure and content of individual sentences and words, newer approaches have the advantage of assessing the text more holistically, assessing high-level features such as cohesion and coherence [11-16]. The Editor could also help users identify whether jargon or acronyms are defined the first time they are used, and potentially incorporate this assessment into the text complexity score. Further work is also needed to establish how these newer assessments relate to the understanding of health information in health literacy priority populations, and to establish how information about coherence and cohesion can be effectively conveyed to users of the tool who are developing health information. Lastly, these assessments are often implied but not explicit in health literacy guidelines, and this additional research could ultimately help refine health literacy guidelines and improve their evidence base.

### Conclusions

The SHeLL Health Literacy Editor provides health services and health information providers with an innovative new tool to improve written health information. The Editor provides objective, immediate feedback on a range of factors, complementing readability with other less widely used and objective assessments such as complex language. The Editor presents health services with a scalable and accessible intervention to address health literacy that staff developing written health information in different settings can easily use. This early prototype has several avenues through which the Editor can be further refined, including expanding the thesaurus and leveraging new machine learning algorithms for assessing the complexity of written text and suggesting alternative phrasing. Ultimately, these efforts seek to build capacity for health information developers to understand health literacy principles and then apply them effectively to educational materials. This systems-based approach has the potential to substantially improve the health literacy environment in our communities.

## Abbreviations

SHeLL: Sydney Health Literacy Lab
SMOG: Simple Measure of Gobbledygook
PEMAT: Patient Education Materials Assessment Tool
